# Sinus Laser-Assisted Closure Under Tumescent Local Anesthesia for Pilonidal Disease: A Pilot Study

**DOI:** 10.7759/cureus.115748

**Published:** 2026-09-03

**Authors:** Gabriela K Pomper, Antun Previsic, Antonija Vrbanic Sutalo, Bozica Lovric, Marin Mamic, Albin Kapetanovic, Ferdinand Slisuric, Ivan Vukoja

**Affiliations:** 1 Faculty of Dental Medicine and Health, University of Osijek, Osijek, HRV; 2 Department of Surgery, General Hospital Požega, Požega, HRV; 3 Department of Anesthesiology, General Hospital Požega, Požega, HRV; 4 Faculty of Dental Medicine and Health Osijek, Josip Juraj Strossmayer University of Osijek, Osijek, HRV; 5 Department of Nursing, University of Applied Sciences Ivanić-Grad, Ivanić Grad, HRV; 6 Department of Emergency Medicine, General Hospital Požega, Požega, HRV; 7 Faculty of Medicine, University of Rijeka, Rijeka, HRV; 8 Department of Internal Medicine, General Hospital Požega, Požega, HRV

**Keywords:** laser therapy, pilonidal sinus, pilonidal sinus disease, pilonidal sinus laser, sinus laser-assisted closure, tumescent local anesthesia, tumescent technique

## Abstract

Objectives: Sinus laser-assisted closure (SiLaC) is a minimally invasive option for management of pilonidal sinus disease (PSD). Tumescent local anesthesia (TLA) may further facilitate outpatient treatment; however, its use in this setting has not been adequately studied. This pilot study aimed to assess the feasibility of SiLaC performed under TLA.

Materials and methods: Between September 2025 and January 2026, 22 consecutive patients with PSD underwent SiLaC under TLA. Demographic characteristics, operative time, total volume of tumescent solution, and calculated lidocaine dose were recorded. Pain was assessed preoperatively, intraoperatively, and on postoperative days 1, 2, 3, and 7 using the Visual Analog Scale (VAS; 0-10). Postoperative complications and outpatient follow-up visits were documented, and early recurrence was assessed at 90 days within a predefined follow-up window of 90 ± 7 days.

Results: The median age was 23 years (range, 18-62 years), and 19 of 22 patients were male. Median operative time was 30 minutes (range, 25-45 minutes), and median tumescent volume was 160 mL (range, 80-280 mL). Median VAS scores were 1 preoperatively (range, 1-2), 2 intraoperatively (range, 1-3), 3 on postoperative day 1 (range, 1-6), 2.5 on day 2 (range, 1-5), 2 on day 3 (range, 1-7), and 1 on day 7 (range, 1-2). Three minor postoperative events occurred: two cases of localized painful swelling and one case of minor bleeding. No anesthesia-related or major complications were observed. All 22 patients completed the 90-day follow-up; no patients were lost to follow-up, and no recurrences, wound healing disorders, or delayed complications were observed in the full evaluable cohort (0/22).

Conclusion: SiLaC performed under TLA appears feasible, well tolerated, and safe for appropriately selected patients in an outpatient setting. Given the small sample size, absence of a control group, and limited follow-up, larger prospective comparative studies with longer follow-up are needed to validate these findings and to determine whether TLA offers advantages over conventional local infiltration anesthesia, particularly in more extensive disease.

## Introduction

Pilonidal sinus disease (PSD)

Standard treatment of PSD traditionally consisted of conventional excisional surgery, including wide excision with primary closure, flap procedures, or healing by secondary intention [1,2]. The introduction of minimally invasive approaches has enabled tissue-sparing treatment options such as sinus laser-assisted closure (SiLaC) [3,4]. Compared with conventional surgery, these techniques are associated with less postoperative pain, faster wound healing, and earlier return to daily activities [5,6], and several studies have reported favorable short- and long-term outcomes [7]. While limited disease can be managed under local anesthesia in an outpatient setting, extensive disease with multiple tracts and cavities may still require inpatient surgery under general anesthesia.

Tumescent local anesthesia (TLA)

TLA represents a specialized form of local anesthesia in which a highly diluted anesthetic solution is infiltrated into the subcutaneous tissue. Originally developed for liposuction [8], its application has since expanded to a wide range of dermatologic and plastic surgical procedures, as well as to vascular, endocrine, and oncologic surgery [9-14].

In conventional infiltration anesthesia, the recommended maximum dose of lidocaine generally ranges from approximately 4 mg/kg to 7 mg/kg when combined with epinephrine [15]. In contrast, substantially higher doses have been reported as well tolerated in the context of TLA because of delayed systemic absorption and distinct pharmacokinetic properties [16-18]. The administration of TLA, nevertheless, requires careful consideration of dose, concentration, treated surface area, vascularity, and patient-specific factors, and the permissible maximum dose remains a subject of discussion [19,20].

While TLA has been extensively investigated in liposuction and dermatologic surgery and more recently in endovenous procedures [9,21,22], its application in proctologic and PSD surgery remains insufficiently explored [23-25]. To our knowledge, TLA has not previously been specifically evaluated as the anesthetic technique for SiLaC. Therefore, we aimed to assess the feasibility, tolerability, and short-term safety of this approach.

## Materials and methods

Study design

In this prospective pilot study, patients undergoing SiLaC under TLA between September 2025 and January 2026 were consecutively enrolled. Inclusion criteria were a confirmed diagnosis of PSD, an indication for laser treatment, and the ability to provide informed consent. Patients with severe multimorbidity, chronic pain syndrome, or imprisonment were excluded. The study protocol was approved by the Ethics Committee of the Požega General County Hospital, Požega, Croatia (approval number: 02-7/3-3/2-2-2025) and conducted in accordance with Good Clinical Practice and the Declaration of Helsinki.

Data collection

Demographic variables included age, sex, body mass index (BMI), smoking status, daily sedentary time, family history of PSD, and regular physical activity. Operative duration and the volume of tumescent solution used were recorded. Subjective pain was assessed preoperatively, intraoperatively, and on postoperative days 1, 2, 3, and 7 using the Visual Analog Scale (VAS; 0 = no pain, 10 = worst imaginable pain). The number of postoperative outpatient follow-up visits and all complications were documented.

Surgical procedure

For local anesthesia, a modified Klein solution consisting of 500 mL normal saline, 20 mL 2% lidocaine, 1 mL epinephrine (1 mg/mL), and 5 mL sodium bicarbonate was prepared. Patients were positioned prone. After sterile preparation and draping, the tumescent solution was administered using an infiltration pump (Figure 1). 

**Figure 1:**
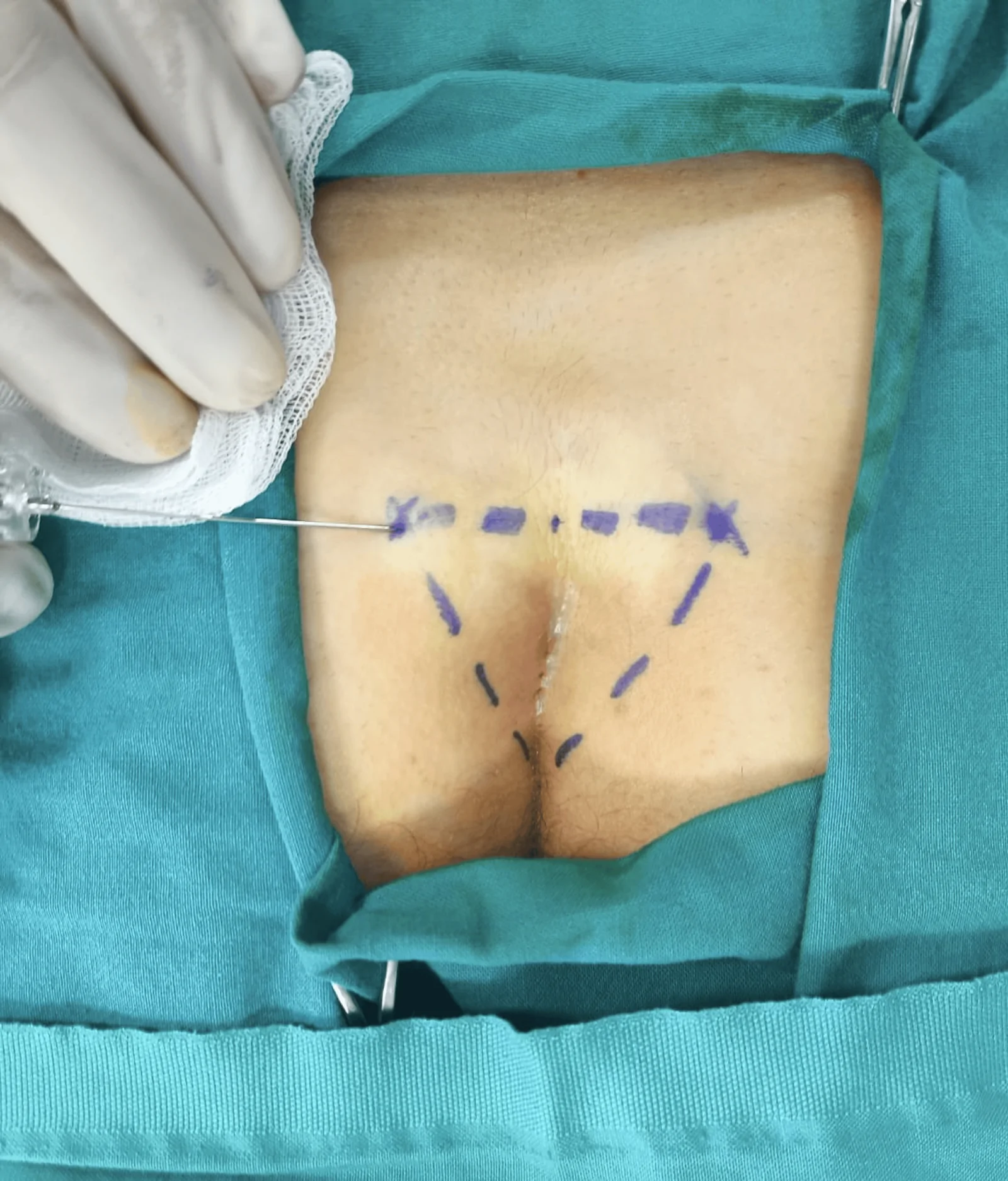
Intraoperative view demonstrating the planned infiltration lines for tumescent local anesthesia surrounding the surgical field. The infiltration is performed along the marked lines while avoiding direct puncture of the pilonidal sinus openings and tracts.

The solution was infiltrated into the subcutaneous tissue circumferentially around the operative field in an approximately triangular pattern surrounding the pilonidal sinus area.

Particular attention was paid to avoiding direct puncture or infiltration of the sinus tracts. Infiltration was continued until the skin and subcutaneous tissues of the operative field were visibly tense and pale/blanched, indicating adequate tumescence and vasoconstriction. 

Following a latency period of approximately 10 minutes, the sinus tract was probed, repeatedly irrigated with a povidone-iodine/saline solution, and mechanically debrided. Laser energy was subsequently applied using a 1470-nm 360° radial-emitting laser fiber connected to a LEONARDO® Mini 1470 nm diode laser system (biolitec biomedical technology GmbH, Jena, Germany). The fiber was advanced to the distal end of each sinus tract and then continuously withdrawn at an approximately constant speed of 1 mm/s while delivering continuous laser energy. All identified tract branches were treated in the same manner. Laser power was adjusted between 8 and 10 W according to the individual anatomy and tissue characteristics. This technique is consistent with the recently published ISoLP position paper on standardized SiLaC technique [26].

Postoperative care

A sterile dry wound dressing was applied at the conclusion of the procedure. Patients were instructed to shower the wound area twice daily and to replace the sterile gauze independently after each cleansing. The first routine postoperative follow-up visit was scheduled for postoperative day 7.

Outcomes and follow-up

The primary feasibility outcomes were: (1) completion of SiLaC under TLA without conversion to general anesthesia or abandonment of the procedure; (2) intraoperative pain measured using the VAS; (3) total tumescent volume and calculated lidocaine dose; and (4) anesthesia-related adverse events. Operative time and the requirement for additional intraoperative tumescent solution were also recorded.

Secondary outcomes included postoperative pain on days 1, 2, 3, and 7; unplanned outpatient visits; and postoperative complications occurring within 30 days. Clinical follow-up was extended to 90 days to identify early recurrence and delayed complications. The 90-day assessment was performed within a predefined window of 90 ± 7 days. Because the follow-up period remained limited, complete healing time and long-term recurrence were not prespecified effectiveness outcomes and were not inferred from the 90-day findings.

Demographic variables included age, sex, BMI, smoking status, daily sedentary time, and regular physical activity. Pain was assessed with a 10-point VAS, where 0 represented no pain and 10 the worst imaginable pain.

Statistical analysis

Because this was a feasibility pilot study without a comparator group and was not powered for hypothesis testing, the analysis was descriptive. Continuous variables are presented as median and range, and categorical variables as absolute number and percentage. Percentages were calculated using the full cohort (n=22) as the denominator. No imputation of missing data was performed; missing baseline information was retained and explicitly reported as "not reported" where applicable. No inferential statistical tests were performed.

## Results

Demographic data

Between September 2025 and January 2026, 22 patients with PSD were enrolled. The median age was 23 years (range, 18-62 years), and most patients were male (19 men, three women). The median BMI was 28.1 kg/m² (range, 20.5-38.8 kg/m²). Thirteen patients were non-smokers, whereas nine were active smokers. Regarding daily physical activity of at least 30 minutes, 11 patients reported participation in sports such as football, seven reported regular walking, one reported regular cycling, and two reported no regular physical activity; physical-activity data were not reported for one patient. The median daily sedentary time was seven hours (range, three to 12 hours) (Table 1).

**Table 1:** Demographic and baseline characteristics Values are presented as n (%) or median (range). Percentages were calculated using the full cohort (n=22). PSD: pilonidal sinus disease

Variable	Value
Number of patients	22
Age, years	23 (18-62)
Sex	
Male	19 (86.4%)
Female	3 (13.6%)
BMI, kg/m²	28.1 (20.5-38.8)
Smoking status	
Non-smoker	13 (59.1%)
Smoker	9 (40.9%)
Physical activity >=30 min/day	
Sports	11 (50.0%)
Walking	7 (31.8%)
Cycling	1 (4.5%)
None	2 (9.1%)
Not reported	1 (4.5%)
Sedentary activity, hours/day	7 (3-12)
Family history of PSD	
Yes	7 (31.8%)
No	12 (54.5%)
Not reported	3 (13.6%)

Perioperative period and pain scores

All 22 procedures were completed under TLA without conversion to general anesthesia or abandonment of the procedure. The median duration of surgery was 30 minutes (range, 25-45 minutes). Median VAS scores were low, with a preoperative median of 1 (range, 1-2) and an intraoperative median of 2 (range, 1-3). The median volume of tumescent solution administered was 160 mL (range, 80-280 mL), corresponding to a median total lidocaine dose of 121.6 mg per patient and a median dose of 1.28 mg/kg (range, 0.49-2.53 mg/kg) (Table 2).

**Table 2:** Perioperative parameters and pain scores Parameters are presented as median (range). VAS: Visual Analog Scale

Parameter	Value
Duration of surgery, minutes	30 (25-45)
Tumescent volume, mL	160 (80-280)
Total lidocaine dose, mg	122 (61-213)
Lidocaine dose, mg/kg	1.28 (0.49-2.53)
VAS preoperative	1 (1-2)
VAS intraoperative	2 (1-3)
VAS day 1	3 (1-6)
VAS day 2	2.5 (1-5)
VAS day 3	2 (1-7)
VAS day 7	1 (1-2)

Postoperative pain and clinical outcomes

Postoperative median VAS scores peaked on the first day after surgery at 3 (range, 1-6), decreased to 2.5 on day 2 (range, 1-5) and 2 on day 3 (range, 1-7), and returned to baseline levels by day 7 at 1 (range, 1-2). Most patients required only one routine outpatient visit, with occasional additional visits when clinically indicated. Two patients experienced postoperative swelling associated with pain, and one patient presented with minor bleeding. These patients also required the highest number of postoperative outpatient visits (up to five). No major complications or anesthesia-related adverse events were recorded.

All 22 patients (100%) completed the 90-day follow-up within the predefined 90 ± 7-day window; no patient was lost to follow-up. No wound healing disorders, recurrences, or delayed complications were observed in the full evaluable cohort at 90 days (recurrence, 0/22 (0%)) (Table 3).

**Table 3:** Postoperative outcomes Values are presented as n (%) or median (range). Percentages were calculated using the full cohort (n=22).

Outcome	Value
Routine outpatient visits	1 (1-5)
Minor complications	3 (13.6%)
Swelling with pain	2 (9.1%)
Minor bleeding	1 (4.5%)
Major complications	0 (0.0%)
Anesthesia-related adverse events	0 (0.0%)

## Discussion

This prospective pilot study demonstrates the feasibility of performing SiLaC for PSD under TLA in an outpatient setting. All 22 procedures were completed without conversion to general anesthesia, intraoperative pain remained low, and no anesthesia-related adverse events were observed. In addition, all patients completed the 90-day follow-up, and no early recurrence was identified. These findings are consistent with the generally favorable perioperative profile reported for minimally invasive laser treatment of PSD [3-7], but they should be interpreted as evidence of feasibility and short-term safety rather than comparative effectiveness because the study did not include a conventional local anesthesia or general anesthesia control group.

TLA offers several practical properties that are relevant to minimally invasive surgery. Infiltration of a large volume of highly diluted local anesthetic provides local analgesia, while epinephrine contributes vasoconstriction, and the fluid itself facilitates tissue separation and hydrodissection [8,10,18]. The pharmacokinetic profile of tumescent lidocaine is characterized by delayed systemic absorption, which explains why substantially higher lidocaine doses have been used in established tumescent techniques than in conventional infiltration anesthesia [15-20]. Reports from liposuction and other tumescent procedures have described the tolerability of substantially higher lidocaine doses when appropriate tumescent technique and patient selection are used [8,15,16,18,19]. In the present cohort, however, the median lidocaine dose was only 1.28 mg/kg, and the maximum recorded dose was 2.53 mg/kg, reflecting the deliberately limited disease burden of the patients included in this pilot.

The broader clinical experience with TLA supports its versatility across different surgical fields. Beyond its original use in liposuction [8], tumescent techniques have been described in dermatosurgery, gynecomastia surgery, cutaneous oncologic surgery, and wide-awake breast surgery [10-14]. Tumescent infiltration is also well established in endovenous interventions [9,21,22], and previous reports have demonstrated its use for cleft-lift, excision, flap procedures, and other proctologic operations [23-25]. These published applications support the biological and practical plausibility of applying TLA to minimally invasive pilonidal surgery [23-25].

Importantly, the patients in the present pilot were intentionally selected from clinical presentations that could also have been treated under conventional local infiltration anesthesia. This stepwise approach was chosen to establish procedural feasibility and tolerability before considering the technique in patients with larger or more complex disease. At our department, disease with more than three sinus tracts and/or a large nidus has generally been treated under general anesthesia, including selected SiLaC procedures. Previous pilonidal studies have shown that TLA can be used for larger excisional and flap-based procedures [23,24], providing a rationale for future evaluation of TLA in more extensive SiLaC cases. However, the present study does not establish that TLA is superior to conventional local anesthesia or that it can replace general anesthesia in extensive disease.

Previous studies of TLA in pilonidal disease have focused on excisional or flap-based procedures rather than laser-assisted tract closure [23,24], whereas reports of SiLaC and related radial laser techniques have primarily addressed technical success, healing, patient recovery, and recurrence without specifically evaluating TLA [3-7]. To our knowledge, the present study is the first prospective evaluation of TLA specifically for SiLaC. The absence of recurrence at 90 days is encouraging but should be regarded only as an early clinical outcome. Recurrence after minimally invasive pilonidal treatment may occur later, and longer-term follow-up is required when treatment effectiveness is assessed [4,7].

The study has several limitations. The sample size was small, there was no comparator group, and the study was designed for feasibility rather than inferential efficacy testing. Patients with more extensive disease were not included, which limits generalizability to the population in whom the potential anesthetic advantages of TLA may be most relevant. Finally, although follow-up was complete, 90 days is insufficient to assess long-term healing and recurrence, particularly because long-term studies of laser treatment for PSD have demonstrated that recurrence assessment requires substantially longer observation [4,7]. A prospective comparative study of TLA versus conventional local infiltration anesthesia, with longer follow-up and predefined disease-complexity strata, is therefore warranted.

## Conclusions

SiLaC under TLA was feasible in all 22 patients in this prospective pilot study, with low perioperative pain, low lidocaine doses, no conversion to general anesthesia, and no anesthesia-related adverse events. Follow-up at 90 ± 7 days was complete for the entire cohort, with no early recurrences or delayed complications. These findings support further evaluation of TLA as an anesthetic strategy for SiLaC but do not establish superiority over conventional local anesthesia. Larger comparative studies with longer follow-up, including patients with more extensive PSD, are required.
